# Structural equation modeling to identify the risk factors of diabetes in the adult population of North India

**DOI:** 10.1186/s41182-018-0104-y

**Published:** 2018-06-25

**Authors:** Jaya Prasad Tripathy, J S Thakur, Gursimer Jeet, Sanjay Jain

**Affiliations:** 1 International Union Against Tuberculosis and Lung Disease, The Union South East Asia Office, New Delhi, India; 2 International Union Against Tuberculosis and Lung Disease, Paris, France; 3 Post Graduate Institute of Medical Education and Research, Chandigarh, India

**Keywords:** Diabetes, epidemiology, STEPS survey, Waist circumference, Family history of diabetes, Structural equation modeling, Blood glucose

## Abstract

**Background:**

A non-communicable disease risk factor survey (based on World Health Organization STEP approach to Surveillance, i.e., WHO-STEPS) was done in the state of Punjab, India in a multistage stratified sample of 5127 individuals. The study subjects were administered the WHO STEPS questionnaire and also underwent anthropometric and biochemical measurements. This study aimed at exploring the risk factors of diabetes using a Structural Equation Modeling (SEM) approach in the North Indian state of Punjab.

**Results:**

Overall prevalence of diabetes mellitus among the study participants was found out to be 8.3% (95% CI 7.3–9.4%). The final SEM had excellent fit considering the model parameters. The following risk factors deemed to have a direct statistically significant effect on blood sugar status: family history of diabetes (4.5), urban residence (3.1), triglycerides (0.46), increasing waist circumference (0.18), systolic blood pressure (0.11), and increasing age (0.05). There are specific indirect effects of alcohol use (1.43, *p* = 0.001), family h/o diabetes (0.844, *p* = 0.001), age (0.156, *p* < 0.001), waist circumference (0.028, *p* = < 0.001) and weekly fruit intake (− 0.009, *p* = 0.034) on fasting blood glucose. Indirect effects of waist circumference, alcohol intake and age on blood sugar levels are mediated by raised blood pressure. Waist circumference mediates the indirect effects of age, family h/o of diabetes, alcohol intake and weekly fruit intake on blood sugar levels. Triglycerides also mediated the indirect effects between age and diabetes.

**Conclusions:**

Family history of diabetes, urban residence, alcohol use, increasing age, and waist circumference are the key variables affecting diabetes status in the Indian population. The results of this study further strengthens the evidence that lifestyle changes in the form of physical activity and healthy diet are required to prevent and control diabetes. Those with family h/o diabetes constitute a high risk group and should be targeted with regular screening and lifestyle intervention package.

## Background

Globally, in 2017 around 425 million adults aged 20–79 years had diabetes mellitus, and this number is expected to rise to 629 million by 2045 [1]. More than three-fourth of the subjects with diabetes belongs to low- and middle-income countries (LMICs). In the South-East Asian region, current estimates indicate that 8.5% of the adult population has diabetes which is likely to increase to 11.1% by 2045. The region has the second highest number of deaths attributable to diabetes among the seven regions with 1.1 million deaths estimated in 2017 [1].

Diabetes is growing at an alarming pace in India due to the rise in various risk factors in both urban and rural areas [2]. India is home to more than 74 million people with diabetes which is second only to China [1]. The burden of diabetes mellitus continues to increase and is primarily driven by nutrition, lifestyle and demographic transitions, unhealthy dietary habits, and physical inactivity, in the context of a stronger genetic predisposition to diabetes [3]. Against this background, a better understanding of the changing epidemiology of diabetes in India is required.

Diabetes mellitus is a condition caused by the complex interplay of various factors simultaneously with some having direct and some having indirect (i.e., mediator) effects. Several risk factors have been associated with type 2 diabetes: age, obesity, high blood pressure, lipid abnormalities, family history of diabetes, unhealthy diet, and physical inactivity [1, 4, 5]. Most available literature used traditional regression models which allow us to treat each covariate in the model as an independent direct effect on diabetes. Very few studies have examined all these factors simultaneously as a network of multiple pathways leading to diabetes [6].

Structural equation modeling (SEM) or path analysis is a very powerful multivariate technique that enables measurement of both direct and indirect effects of variables and incorporate models with multiple dependent variables by using several regression equations simultaneously [7]. Thus, the present study employed SEM to test a hypothesized model of variables affecting diabetes status in the North Indian adult population using data from a large representative household survey.

## Methods

### Study design

This study analyses data from a large household survey conducted in the state of Punjab, India using WHO-STEPwise approach to surveillance (STEPS) approach [8].

### Study setting

The survey was carried out in Punjab, which is a prosperous state in the northern part of India bordering Pakistan with a population of 27 million according to 2011 national census. It ranks higher than most other states in terms of Human Development Index with a per capita income twice that of the national average [9, 10].

### Study sampling strategy

A state-wide non-communicable disease (NCD) risk factor survey based on WHO-STEPS approach was undertaken in Punjab in 2014–2015. The survey adopted a multistage stratified sampling approach using the 2011 census sampling frame. A three-stage design was employed in urban areas whereas in rural areas a two-stage sampling design was followed. A total of 100 primary sampling units (PSUs) were selected (60 villages from rural areas and 40 Census Enumeration Blocks from urban areas) by probability proportional to size (PPS) method. From each selected PSU, 54 households were selected using systematic random sampling. The ultimate sampling units were the households and one individual in the age group of 18–69 years residing in the selected household was selected using the KISH method. The details of the methodology can be found in another paper [11].

### Data collection instrument

A culturally adapted, *Punjabi* (local language) translated and pre-tested version of the WHO STEPS questionnaire (version 3.1) was used with minor adaptations [12]. As part of the household survey, sociodemographic and behavioral information on tobacco and alcohol use, diet, physical activity, history of chronic diseases, family history of chronic conditions, health screening, and health care expenditure were collected in step 1. Physical measurements such as height, weight, blood pressure, and waist circumference were done in step 2. Biochemical tests were conducted to measure fasting blood glucose, total cholesterol, and triglycerides in step 3.

### Data collection and operational definitions used

A team of trained investigators collected the survey data. SECA adult portable stadiometer was used to measure height after removing shoes, socks, slippers, and any head gear. It was measured in centimeters up to 0.1 cm. SECA digital weighing scale was used to measure weight of the individuals. The scale was regularly calibrated against a standard weight. The participants were asked to remove footwear and socks, and weight was recorded in kilograms up to 0.1 kg. Waist circumference was measured using a SECA constant tension tape to the nearest 0.1 cm at the level of the midpoint between the inferior margin of the last rib and the iliac crest in the mid-axillary plane. The measurement was taken at the end of a normal expiration with the arms relaxed at the sides.

One serving of vegetable was considered to be one cup of raw green leafy vegetables or 1/2 cup of other vegetables (cooked or chopped raw). One serving of fruit was considered to be one medium size piece of apple, banana, or orange; 1/2 cup of chopped, canned fruit; or 1/2 cup of fruit juice.

Physical activity was assessed using the Global Physical Activity Questionnaire (GPAQ), which has been developed by the World Health Organization. This questionnaire assesses physical activity behavior in three different domains: work, transport, and during leisure time. Activities are classified into three intensity levels: vigorous, moderate, and light based on the physical effort it requires. Participants were classified as sufficiently active who exceed the minimum duration of physical activity per week recommended by WHO, i.e., 150 min of moderate intensity physical activity or 75 min of vigorous intensity physical activity or an equivalent combination of moderate- and vigorous-intensity physical activity achieving at least 600 MET-minutes per week with each activity performed in bouts of at least 10-min duration [13]. Body mass index (BMI) was calculated as weight in kilograms/height in meters squared. Show cards (pictorial, adapted to the local context) were used to explain to the participants the type of physical activity, servings of fruits and vegetables, and salty food intake. Obesity was defined as a BMI ≥ 27.5 kg/m^2^ for both genders (based on the World Health Organization Expert Consultation for Asian populations) [14]. Abdominal obesity was defined as a waist circumference ≥ 90 cm for men and ≥ 80 cm for women [15].

For blood pressure measurement, electronic equipment (OMRON HEM 7120, Omron Corporation, Kyoto, Japan) was used. After resting for 5 min, blood pressure was recorded in the sitting position in the right arm supported at the level of the heart. Three blood pressure measurements were taken at 3 min interval each. The final reading was recorded as the average of last two readings.

Biochemical measurements (step 3): every alternate individual (50%) of the initial sample was subjected to biochemical assessment. For blood glucose, dry chemistry method was used by blood glucose measurement device (Optium H, Freestyle). For lipid profile, i.e., cholesterol and triglycerides measurements, blood samples were drawn on individuals after 10–12 h fasting. 5 ml of venous blood was taken in sitting position, was centrifuged immediately to separate serum, and was transferred under cold chain condition to the Central Reference Laboratory of Department of Biochemistry, Post Graduate Institute of Medical Education and Research, Chandigarh, India which is a tertiary medical care institute.

Laboratory measurement of total cholesterol and triglyceride was made on Modular P 800 autoanalyzer (Roche Diagnostics, Germany) using commercially available kits (Roche Diagnostics, Germany). Hypertriglyceridemia was defined as serum triglyceride levels ≥ 150 mg/dl (≥ 1.7 mmol/l) [16].

### Sample size

Taking the estimated prevalence of physical activity as 50%, 5% margin of error and 95% confidence interval, a design effect of 1.5, a sample size of 4609 was derived which was adequate to present results by two age groups (18–44, 45–69), both sexes (male, female) and residence (urban, rural). Assuming a response rate of 85%, sample size was raised to 5400 for this study. Every second individual was subjected to step 3, i.e., biochemical assessment. Out of 2700 respondents eligible for step 3, 2499 (93%) gave consent to blood sampling for biochemical assessment.

#### Statistical analysis

**Fig. 1:**
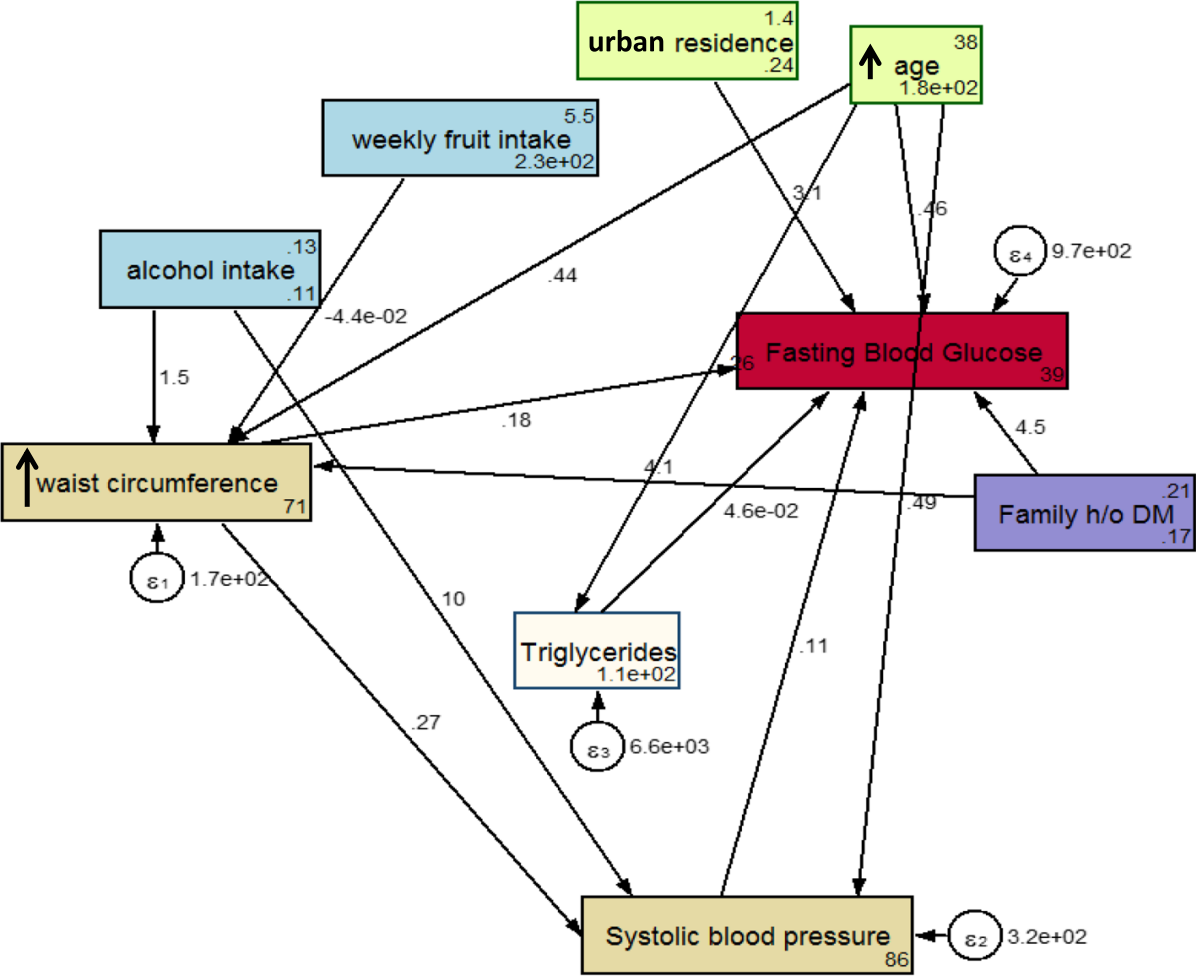
SEM model of risk factors for diabetes in the STEPS survey in 2015 in Punjab, India among adults aged 18–69 years. Footnote: SEM = structural equation modeling; box indicates observed variable; straight line with one arrowhead denotes direct effect

The conceptual a priori model that specifies the relations among variables operationalized in this study is based on the model proposed by Bardenheier et al [17]. We used structural equation modeling with *path analysis*, which includes the direct and indirect effects of factors previously reported to be associated with diabetes (Fig. 1). Direct effects are depicted as an arrow originating from an independent variable (exposure) leading and pointing to a dependent variable (outcome). For example, see the arrow between waist circumference and systolic blood pressure. An indirect effect is not only depicted as a mediating variable having an arrow pointing to it from an independent variable but also pointing to yet another dependent variable. For example, waist circumference mediates the effect of alcohol intake on blood sugar levels. A confounder, according to the use of these arrows, is depicted as a variable with direct effects on both the exposure and the dependent variable.

In this study, we report standardized path coefficients, their standard errors and *p* values. As indices of the models’ statistical fit to the data, we used standard criteria, including comparative fit index (CFI) > 0.90, root mean square error of approximation (RMSEA) < 0.08, and the standardized root mean square residual (SRMSR) < 0.06. Model building and estimation was done using STATA/IC version 12 (StataCorp LP, USA).

#### Variables assessed in SEM

We selected the sociodemographic, behavioral, anthropometric, and metabolic variables to be included in our SEM based on a literature review of previous theoretical models of diabetes. We assessed 13 variables including age (in years), sex, residence (rural and urban), highest level of education (no formal schooling, up to primary schooling, up to secondary schooling, up to higher secondary, graduate, and postgraduate degree), marital status (never married, currently married, divorced/separated/widowed), current smoking status (was defined as positive if the subject smoked in the last 30 days), current alcohol status (was defined as positive if the subject consumed alcohol in the last 365 days), BMI in kg/m^2^, family history of diabetes (yes or no), waist circumference (in centimeters), systolic blood pressure (in mm of Hg), fasting blood glucose (in mg/dl), triglycerides (in mg/dl), total cholesterol (in mg/dl), physical activity (self-reported hours of vigorous-intensity work and sports activities/recreation and hours of walking/cycling), and weekly fruit/vegetable intake (number of servings of fruits/vegetable). We have reported pathways only for statistically significant standardized path coefficients at *p* < 0.05 level.

#### Ethics approval

The Institute Ethics Committee of Post Graduate Institute of Medical Education and Research, Chandigarh approved the study (reference number P-727, dated 21 July 2014). Informed written consent was taken from all participants.

## Results

### Prevalence of diabetes

Out of 2499, a total of 2495 study participants were included (four excluded due to missing data). Overall prevalence of diabetes mellitus among the study participants was found out to be 8.3% (95% CI 7.3–9.4%). Individuals with diabetes were more likely to be between 45 and 69 years (18%, *p* = 0.001), belonging to general (9.3%) and other backward caste (10.2%, *p* = 0.05), divorced/separated/widowed (14.9%, *p* = 0.001), hypertensive (14.3%, p = 0.001), obese (14.4%, *p* = 0.001), with family history of diabetes (11.9%, *p* = 0.001), have poor physical activity (8.6%, *p* = 0.008), and hypertriglyceridemia (10.8%, *p* = 0.006). Table 1.

**Table 1:** Characteristics of study participants and the prevalence of diabetes by sociodemographic and clinical factors

Characteristics	Total, *N*	Diabetes, *n* (%)	*p* value
Total	2495	207 (8.3)	
Age group			0.001
18–24 years	430	6(1.4)	
25–44 years	1289	61(4.7)	
45–69 years	776	140(18.0)	
Gender			0.9
Male	907	76(8.4)	
Female	1588	131(8.2)	
Residence			0.11
Rural	1529	116(7.6)	
Urban	966	91(9.4)	
Social group			0.05
SC	954	61(6.4)	
Other backward caste	342	35(10.2)	
General	1155	107(9.3)	
Educational status			0.07
Illiterate	575	51(8.9)	
Up to primary education	599	63(10.3)	
Up to secondary education	1065	74(6.9)	
Higher education	256	19(7.4)	
Marital status			0.001
Never married	413	5(1.2)	
Currently married	1869	172(9.2)	
Separated/divorced/widowed	195	29(14.9)	
Current smoking^a^			0.7
Yes	101	8(7.9)	
No	2394	199(8.3)	
Current alcohol use^b^			0.1
Yes	386	40(10.4)	
No	2109	167(7.9)	
Hypertension^c^			0.001
Yes	1031	147(14.3)	
No	1464	60(4.1)	
> = 5 servings of fruits and vegetables daily^d^			0.4
Yes	109	10(9.2)	
No	2386	197(8.3)	
Obesity^e^			0.001
Yes	1830	96(14.4)	
No	665	111(6.1)	
Family history of diabetes			0.001
Yes	537	64(11.9)	
No	1925	142(7.4)	
Family history of raised cholesterol			0.9
Yes	126	11(8.7)	
No	2336	195(8.3)	
Abdominal obesity^f^			0.001
Yes	1415	165(11.7)	
No	1080	42(3.9)	
Hypertriglyceridemia^g^			0.006
Yes	656	136(7.4)	
No	1839	71(10.8)	
Physical activity^h^			0.008
Above minimal recommended	140	04 (3.0)	
Below minimal recommended	2355	203(8.6)	

*SC* scheduled caste

^a^One who smoked tobacco in the last 30 days

^b^One who has consumed alcohol in the last year

^c^SBP ≥ 140 and/or DBP ≥ 90 or currently on medication

^d^One serving of vegetable was considered to be one cup of raw green leafy vegetables or 1/2 cup of other vegetables (cooked or chopped raw). One serving of fruit was considered to be one medium size piece of apple, banana or orange, 1/2 cup of chopped, canned fruit, or 1/2 cup of fruit juice

^e^Body mass index ≥ 27.5 kg/m^2^

^f^≥90 cm for males and ≥ 80 cm for females

^g^Serum triglyceride > 150 mg/dl

^h^Minimum duration of physical activity per week recommended by WHO as 150 min of moderate intensity physical activity or 75 min of vigorous intensity physical activity or an equivalent combination of moderate- and vigorous-intensity physical activity achieving at least 600 MET-minutes per week with each activity performed in bouts of at least 10-min duration

### SEM model

The final model had excellent fit, and the fully standardized path coefficients are presented in Fig. 1. Non-significant paths were removed and a few additional paths were added to improve model fit. Specifically, education, marital status, BMI, physical activity, smoking status, and serum cholesterol were dropped from the model for better fit. Table 2 shows the coefficients and standard error of the direct and indirect effects of variables on diabetes status. The goodness-of-fit statistics of the model are shown in Table 3.

**Table 2:** Coefficient and standard error of the final SEM model of risk factors for diabetes in the STEPwise approach to surveillance 2015 survey—Punjab, India among adults aged 18–69 years

Variables	Coefficient	Standard error	*p* value	95% CI lower upper	
Direct effects					
Fasting blood glucose					
Waist circumference	0.177	0.051	0.001	0.076	0.278
Systolic blood pressure	0.107	0.035	0.003	0.038	0.177
Triglyceride	0.046	0.008	< 0.001	0.030	0.062
Alcohol use	0	(No path)			
Weekly fruit intake	0	(No path)			
Age	0.464	0.056	< 0.001	0.353	0.574
Urban residence	3.124	1.337	0.020	0.502	5.746
Family h/o diabetes	4.473	1.593	0.005	1.350	7.597
Waist circumference					
Alcohol use	1.521	0.605	0.05	0.305	2.764
Weekly fruit intake	−0.044	0.018	0.01	− 0.079	− 0.009
Age	0.441	0.020	< 0.001	0.401	0.481
Family h/o diabetes	4.104	0.655	< 0.001	2.819	5.389
Triglycerides					
Age	0.264	0.127	0.03	0.015	0.513
Systolic blood pressure					
Waist circumference	0.266	0.028	< 0.001	0.210	0.321
Alcohol use	10.402	1.100	< 0.001	8.245	12.559
Weekly fruit intake	0	(No path)			
Age	0.491	0.030	< 0.001	0.431	0.551
Family h/o diabetes	0	(No path)			
Indirect effects					
Fasting blood glucose					
Waist circumference	0.028	0.003	< 0.001	0.022	0.034
Systolic blood pressure	0	(No path)			
Triglyceride	0	(No path)			
Alcohol use	1.430	0.428	0.001	0.591	2.268
Weekly fruit intake	−0.009	0.004	0.034	−0.017	−0.001
Age	0.156	0.029	< 0.001	0.098	0.213
Urban residence	0	(No path)			
Family h/o diabetes	0.844	0.247	0.001	0.359	1.329
Waist circumference					
Alcohol use	0	(No path)			
Weekly fruit intake	0	(No path)			
Age	0	(No path)			
Family h/o diabetes	0	(No path)			
Triglycerides					
Age	0	(No path)			
Systolic blood pressure					
Waist circumference	0	(No path)			
Alcohol use	0.404	0.216	0.061	− 0.018	0.827
Weekly fruit intake	− 0.012	0.005	0.016	− 0.021	− 0.002
Age	0.117	0.014	< 0.001	0.090	0.144
Family h/o diabetes	1.09	0.210	< 0.001	0.679	1.501
Total effects					
Fasting blood glucose					
Waist circumference	0.206	0.051	< 0.001	0.105	0.306
Systolic blood pressure	0.107	0.035	0.003	0.038	0.177
Triglyceride	0.046	0.008	< 0.001	0.030	0.062
Alcohol use	1.430	0.428	0.001	0.591	2.268
Weekly fruit intake	− 0.009	0.004	0.034	− 0.017	− 0.001
Age	0.619	0.049	< 0.001	0.522	0.716
Urban residence	3.124	1.338	0.02	0.502	5.746
Family h/o diabetes	5.317	1.586	0.001	2.208	8.427
Waist circumference					
Alcohol use	1.521	0.655	0.05	0.234	2.915
Weekly fruit intake	− 0.044	0.018	0.013	− 0.079	− 0.009
Age	0.441	0.020	< 0.001	0.401	0.481
Family h/o diabetes	4.104	0.656	< 0.001	2.819	5.389
Triglycerides					
Age	0.264	0.127	0.038	0.015	0.513
Systolic blood pressure					
Waist circumference	0.266	0.028	< 0.001	0.210	0.321
Alcohol use	10.806	1.120	< 0.001	8.611	13.001
Weekly fruit intake	− 0.012	0.005	0.016	− 0.021	− 0.002
Age	0.608	0.028	< 0.001	0.552	0.664
Family h/o diabetes	1.090	0.210	< 0.001	0.679	1.501

*SEM* structural equation modeling, *SEM model* endogenous variables are waist circumference, systolic blood pressure, triglyceride, fasting blood glucose; exogenous variables are alcohol use, weekly fruit intake, place of residence, age, family history, education and occupation, *CI* confidence interval

**Table 3:** Goodness-of-fit statistics for the structural equation model

Goodness-of-fit statistics	Value
Root mean squared error of approximation	0.04, 90% CI (0.03–0.05)
pclose	0.82, < 0.05
Comparative fit index	0.93
Tucker-Lewis index	0.90
Standardized root mean squared residual	0.02
Coefficient of determination	0.37

The following risk factors deemed to have a direct statistically significant effect on blood sugar status: family history of diabetes (4.5), urban residence (3.1), increasing age (0.05) and waist circumference (0.18), systolic blood pressure (0.11), and triglycerides (0.46) (Figure 1 and Table 2). There are specific indirect effects of waist circumference (0.028, *p* = < 0.001), alcohol use (1.43, *p* = 0.001), weekly fruit intake (− 0.009, *p* = 0.034), increasing age (0.156, *p* < 0.001), and family h/o diabetes (0.844, *p* = 0.001) on fasting blood glucose (Table 2).

**Fig. 2:**
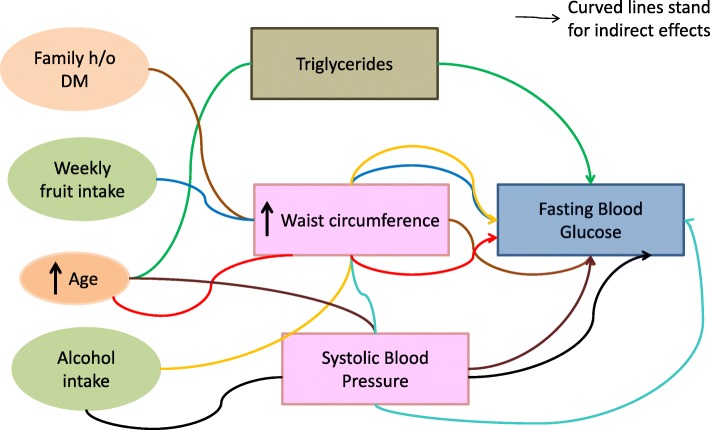
Indirect effects of risk factors for diabetes in the STEPS survey in 2015 in Punjab, India among adults aged 18–69 years. Footnote: ellipse indicates latent, unobservable constructs; box indicates observed variable; curved line with one arrowhead denotes indirect effect

Waist circumference, alcohol intake, and increasing age impact blood sugar levels mediated by raised blood pressure. Waist circumference mediates the indirect effects of age, family h/o of diabetes, alcohol intake, and weekly fruit intake on blood sugar levels. Triglycerides also mediate the indirect effect of age on diabetes status (Fig. 2).

## Discussion

This is the first study in India to use SEM to analyze non-modifiable and modifiable sociodemographic, behavioral, and metabolic determinants of diabetes in India. The following risk factors were found to have a direct statistically significant effect on blood sugar status: family history of diabetes, urban residence, increasing age and waist circumference, blood pressure, and triglycerides. This model also found that there are specific indirect effects of waist circumference, alcohol use, weekly fruit intake, age, and family h/o diabetes on fasting blood glucose.

SEM is a second generation multivariate method that allows causal modeling of diseases with a complex interplay of several factors resulting in measurement of direct and indirect effects and performing models with multiple dependent variables which are inter-related. This methodology fits well in a chronic disease setting where several factors have direct and indirect relationships with the disease through mediator variables forming a web of causation model.

The prevalence of diabetes in the study was found to be 8.3% which is slightly lower than the figure in another recent large nationally representative survey (INDIAB study) which reported prevalence to be 10% in the same state of India [5]. Place of residence has a direct association with diabetes status supported by other studies which show that the prevalence of diabetes continues to be higher in urban areas than in rural areas [5, 18, 19], although some studies show no urban-rural gap [4].

Overall, family h/o diabetes had the strongest effect on the risk of the disease, both direct and indirect. This finding is well supported by the results of the INDIAB study in both rural and urban areas [5]. Family h/o diabetes also indirectly affected diabetes status through raised waist circumference. Thus, those with family h/o diabetes constitute a high risk group requiring a package of services which includes regular screening for blood glucose and a lifestyle behavioral intervention package in terms of healthy diet and physical activity.

Raised triglyceride level is a common dyslipidemic feature accompanying type 2 diabetes. It is one of five accepted criteria for defining individuals at high risk for cardio-metabolic diseases including 2 diabetes, termed the “metabolic syndrome” [16, 20, 21]. The present study, besides an independent effect, also showed that triglyceride levels mediate the effect of age on diabetes as triglyceride levels increase with age [22].

Body fat distribution (excess abdominal fat) is associated with an increased risk of cardio-metabolic diseases such as diabetes, hypertension, dyslipidemia, and coronary heart disease. Waist circumference is often used as a surrogate marker of abdominal fat mass, because it correlates with abdominal fat mass [23, 24]. The present study showed that waist circumference not only had a direct effect on diabetes but also mediated the indirect effects of age, family h/o of diabetes, alcohol intake, and weekly fruit intake on blood sugar levels, thereby strengthening the evidence. The study also adds to the evidence base which states that waist circumference is a stronger predictor of diabetes than BMI, apart from other cardio-metabolic diseases [24]. It provides a unique indicator of body fat distribution, which can identify patients who are at increased risk for diabetes and other obesity-related cardio-metabolic disease, above and beyond the measurement of BMI. Waist circumference will help clinicians determine which patients should be evaluated for the presence of cardio-metabolic risk factors, such as dyslipidemia, raised blood pressure and hyperglycemia and also response to lifestyle behavioral practices. Thus, we recommend that measuring waist circumference measurement should be part of the routine clinical care guidelines.

Although previous studies in India did not find alcohol as an independent risk factor for diabetes [4, 5], the present study showed that the effect of alcohol intake on diabetes was mediated through waist circumference and blood pressure. According to the existing evidence in medical literature, alcohol intake may be a risk factor for obesity or rise in waist circumference [25–27], though contradictory findings do exist [28–30]. Also, several possible mechanisms have been proposed to establish the association between alcohol consumption and hypertension which are described elsewhere [31].

### Strengths and limitations

The strengths of the study include a large multistage stratified community-based sample, high participant response rate, use of standardized STEPS questionnaire, and adherence to STROBE (Strengthening the Reporting of Observational Studies in Epidemiology) guidelines for reporting the findings of the study [32]. The study also employed a robust statistical technique, i.e., SEM which allows measurement of both direct and indirect effects of variables and incorporate models with multiple dependent variables by using several regression equations simultaneously.

There were a few limitations in this study. First, the main outcome measure, i.e., fasting blood glucose which defined diabetes status was assessed at one single point in time when in reality it can naturally fluctuate. Second, capillary blood glucose estimation was done in place of the ideal venous plasma glucose estimation due to logistic constraints such as non-availability of quality-controlled laboratories, storage and transport of blood specimens, varied methods of estimation, and poor compliance to venous blood collection due to its invasive nature. However, it has been reported that capillary blood collection is a feasible and valid alternative to venous blood collection for screening in large epidemiological studies [33, 34]. Third, the cross-sectional nature of the survey prevents us from making causal inferences about the outcome.

## Conclusions

Family history of diabetes, urban residence, alcohol use, increasing age, and waist circumference are the key variables affecting diabetes status in the Indian population. The results of this study further strengthens the evidence that lifestyle changes in the form of physical activity and healthy diet are required to prevent and control diabetes. Those with family h/o diabetes constitute a high risk group and should be targeted with a regular screening and lifestyle intervention package.

## Abbreviations

BMI: Body mass index
LMICs: Low- and middle-income countries
NCD: Non-communicable disease
PPS: Probability proportional to size
PSU: Primary sampling unit
SEM: Structural equation modeling
STROBE: Strengthening the Reporting of Observational Studies in Epidemiology

## References

[1] International Diabetes Federation (2017). IDF Diabetic Atlas 8th Edition.

[2] Tripathy JP, Thakur JS, Jeet G, Chawla S, Jain S, Prasad R (2016). Urban rural differences in diet, physical activity and obesity in India: are we witnessing the great Indian equalisation? Results from a cross-sectional STEPS survey. BMC Public Health.

[3] Mohan V (2004). Why are Indians more prone to diabetes?. J Assoc Physicians India.

[4] Tripathy JP, Thakur JS, Jeet G, Chawla S, Jain S, Pal A (2017). Prevalence and risk factors of diabetes in a large community-based study in North India: results from a STEPS survey in Punjab, India. Diabetol Metab Syndr.

[5] Anjana RM, Deepa M, Pradeepa R, Mahanta J, Narain K, Das HK (2017). Prevalence of diabetes and prediabetes in 15 states of India: results from the ICMR-INDIAB population-based cross-sectional study. Lancet Diabetes Endocrinol.

[6] Roman-Urrestarazu A, Ali FMH, Reka H, Renwick MJ, Roman GD, Mossialos E (2016). Structural equation model for estimating risk factors in type 2 diabetes mellitus in a middle eastern setting: evidence from the STEPS Qatar. BMJ Open Diabetes Res Care.

[7] Karimimalayer M, Alavifar A, Anuar MK (2012). Structural equation modeling VS multiple regression the first and second generation of multivariate techniques. Eng Sci Technol An Int J.

[8] World Health Organization. The WHO STEPwise approach to Surveillance of noncommunicable diseases (STEPS). Geneva; 2003. Available from: http://www.who.int/ncd_surveillance/en/steps_framework_dec03.pdf.

[9] Government of Punjab, India. 2016 [cited 2016 Oct 18]. Available from: http://punjab.gov.in/.

[10] Mukherjee S, Chakraborty D, Sikdar S. Three decades of human development across Indian states: inclusive growth or perpetual disparity? New Delhi; 2014. Available from: http://www.nipfp.org.in/media/medialibrary/2014/06/WP_2014_139.pdf.

[11] Thakur JS, Jeet G, Pal A, Singh S, Singh A, Deepti SS (2016). Profile of risk factors for non-communicable diseases in Punjab, Northern India: results of a state-wide STEPS survey. PLoS One.

[12] World Health Organization. The WHO STEPwise approach to surveillance of noncommunicable diseases (STEPS). Geneva; 2003. [cited 2016 Aug 30]. Available from: http://www.who.int/ncd_surveillance/en/steps_framework_dec03.pdf.

[13] World Health Organization. Global Physical Activity Questionnaire (GPAQ) Analysis Guide. Geneva; 2012. [cited 2017 Apr 5]. Available from: http://www.who.int/chp/steps/resources/GPAQ_Analysis_Guide.pdf.

[14] WHO Expert Consultation (2004). Appropriate body-mass index for Asian populations and its implications for policy and intervention strategies. Lancet.

[15] Regional Office for the Western Pacific (WPRO) World Health Organization (2000). International Association for the Study of Obesity and the International Obesity Task Force. The Asia Pacific perspective: redefining obesity and its treatment.

[16] Expert Panel on Detection, Evaluation, and Treatment of High Blood Cholesterol in Adults (2001). Executive summary of the third report of the National Cholesterol Education Program (NCEP) expert panel on detection, evaluation, and treatment of high blood cholesterol in adults (Adult Treatment Panel III). JAMA.

[17] Bardenheier BH, Bullard KM, Caspersen CJ, Cheng YJ, Gregg EW, Geiss LS (2013). A novel use of structural equation models to examine factors associated with prediabetes among adults aged 50 years and older: National Health and Nutrition Examination Survey 2001-2006. Diabetes Care.

[18] Deepa M, Anjana RM, Manjula D, Narayan KMV, Mohan V (2011). Convergence of prevalence rates of diabetes and cardiometabolic risk factors in middle and low income groups in urban India: 10-year follow-up of the Chennai urban population study. J Diabetes Sci Technol.

[19] Mohan V, Mathur P, Deepa R, Deepa M, Shukla DK, Menon GR (2008). Urban rural differences in prevalence of self-reported diabetes in India—the WHO–ICMR Indian NCD risk factor surveillance. Diabetes Res Clin Pract.

[20] Ginsberg HN, Zhang Y-L, Hernandez-Ono A (2005). Regulation of plasma triglycerides in insulin resistance and diabetes. Arch Med Res.

[21] Alberti KGM, Zimmet P, Shaw J, IDF Epidemiology Task Force Consensus Group (2005). The metabolic syndrome? a new worldwide definition. Lancet.

[22] Tripathy JP, Thakur JS, Jeet G, Chawla S, Jain S, Pal A, Prasad R. Burden and risk factors of dyslipidemia-results from a STEPS survey in Punjab India. Diabetes Metab Syndr. 2017;11 Suppl 1:S21–S27. 10.1016/j.dsx.2016.08.015 27595388

[23] Pouliot MC, Després JP, Lemieux S, Moorjani S, Bouchard C, Tremblay A (1994). Waist circumference and abdominal sagittal diameter: best simple anthropometric indexes of abdominal visceral adipose tissue accumulation and related cardiovascular risk in men and women. Am J Cardiol.

[24] Klein S, Allison DB, Heymsfield SB, Kelley DE, Leibel RL, Nonas C, et al. Waist circumference and cardiometabolic risk: a consensus statement from shaping America's health: Association for Weight Management and Obesity Prevention; NAASO, the Obesity Society; the American Society for Nutrition; and the American Diabetes Association. Diabetes Care. 2007;30(6):1647–52. 10.2337/dc07-9921 17360974

[25] Vadstrup ES, Petersen L, Sorensen TIA, Gronbaek M (2003). Waist circumference in relation to history of amount and type of alcohol: results from the Copenhagen City Heart Study. Int J Obes.

[26] Mozaffarian D, Hao T, Rimm EB, Willett WC, Hu FB (2011). Changes in diet and lifestyle and long-term weight gain in women and men. N Engl J Med.

[27] French MT, Norton EC, Fang H, Maclean JC (2009). Alcohol consumption and body weight. Health Econ.

[28] Liu S, Serdula MK, Williamson DF, Mokdad AH, Byers T (1994). A prospective study of alcohol intake and change in body weight among US adults. Am J Epidemiol.

[29] Pajari M, Pietilainen KH, Kaprio J, Rose RJ, Saarni SE (2010). The effect of alcohol consumption on later obesity in early adulthood—a population-based longitudinal study. Alcohol Alcohol.

[30] Tolstrup JS, Halkjaer J, Heitmann BL, Tjønneland AM, Overvad K, Sørensen TIA (2008). Alcohol drinking frequency in relation to subsequent changes in waist circumference. Am J Clin Nutr.

[31] Husain K, Ansari RA, Ferder L (2014). Alcohol-induced hypertension: mechanism and prevention. World J Cardiol.

[32] von Elm E, Altman DG, Egger M, Pocock SJ, Gøtzsche PC, Vandenbroucke JP (2007). The strengthening the reporting of observational studies in epidemiology (STROBE) statement: guidelines for reporting observational studies. Bull World Health Organ.

[33] Kruijshoop M, Feskens EJM, Blaak EE, de Bruin TWA (2004). Validation of capillary glucose measurements to detect glucose intolerance or type 2 diabetes mellitus in the general population. Clin Chim Acta.

[34] Priya M, Mohan Anjana R, Pradeepa R, Jayashri R, Deepa M, Bhansali A (2011). Comparison of capillary whole blood versus venous plasma glucose estimations in screening for diabetes mellitus in epidemiological studies in developing countries. Diabetes Technol Ther.

